# Global Profiling of Alternative Splicing Events and Gene Expression Regulated by hnRNPH/F

**DOI:** 10.1371/journal.pone.0051266

**Published:** 2012-12-17

**Authors:** Erming Wang, Vahid Aslanzadeh, Filomena Papa, Haiyan Zhu, Pierre de la Grange, Franca Cambi

**Affiliations:** 1 Department of Neurology, University of Kentucky, Lexington, Kentucky, United States of America; 2 Department of Biotechnology, Research Institute of Physiology and Biotechnology, University of Zanjan, Zanjan, Iran; 3 GenoSplice technology, Hôpital Saint-Louis, Paris, France; University of Iowa, United States of America

## Abstract

In this study, we have investigated the global impact of heterogeneous nuclear Ribonuclear Protein (hnRNP) H/F-mediated regulation of splicing events and gene expression in oligodendrocytes. We have performed a genome-wide transcriptomic analysis at the gene and exon levels in Oli-neu cells treated with siRNA that targets hnRNPH/F compared to untreated cells using Affymetrix Exon Array. Gene expression levels and regulated exons were identified with the GenoSplice EASANA algorithm. Bioinformatics analyses were performed to determine the structural properties of G tracts that correlate with the function of hnRNPH/F as enhancers vs. repressors of exon inclusion. Different types of alternatively spliced events are regulated by hnRNPH/F. Intronic G tracts density, length and proximity to the 5′ splice site correlate with the hnRNPH/F enhancer function. Additionally, 6% of genes are differently expressed upon knock down of hnRNPH/F. Genes that regulate the transition of oligodendrocyte progenitor cells to oligodendrocytes are differentially expressed in hnRNPH/F depleted Oli-neu cells, resulting in a decrease of negative regulators and an increase of differentiation-inducing regulators. The changes were confirmed in developing oligodendrocytes *in vivo*. This is the first genome wide analysis of splicing events and gene expression regulated by hnRNPH/F in oligodendrocytes and the first report that hnRNPH/F regulate genes that are involved in the transition from oligodendrocyte progenitor cells to oligodendrocytes.

## Introduction

hnRNPH and F control alternatively spliced events (ASE) by binding to G tracts positioned in close proximity to the 5′ or 3′ splice sites (ss), with G triplets being the basic recognition motif [1], [2], [3], [4], [5]. hnRNPH and F can either enhance or inhibit the alternatively spliced exon and the magnitude of the effect is dependent on the length of the G tracts, the intronic vs. exonic position and the strength of the 5′ ss [6], [7], [8], [9], [10]. We have shown that hnRNPH/F regulate the major myelin proteolipid protein (PLP)/DM20 ratio predominantly by enhancing the selection of the DM20 5′ splice site through long G tracts positioned in exon 3B immediately downstream of the DM20 5′ss [11], [12], [13]. Unlike other ASEs, hnRNPH and F exert a novel synergistic regulation of the PLP alternatively spliced event and their function is not redundant [12].

The alternative splicing of PLP is a differentiation dependent event in the oligodendrocytes (OL), the myelin producing cells of the central nervous system (CNS). Endogenous hnRNPH and F expression is high in oligodendrocyte progenitor cells (OPC) and decreases in differentiated OL *in vitro* at the time when the PLP/DM20 ratio increases [12]. Furthermore, siRNA-mediated knock down of hnRNPH/F increases the PLP/DM20 ratio in the oligodendrocyte cell line, Oli-neu cells [12]. The down regulation of hnRNPH/F is temporally related to the transition of oligodendrocyte progenitor cells to differentiated OL, suggesting that hnRNPH/F may contribute broadly to differentiation-induced changes in gene splicing and expression that occur as part of the OL differentiation program.

Many excellent genomewide studies have characterized the role of G tracts in splicing [6], [7], [14]. A global analysis of genome wide hnRNPH/F mediated regulation of alternative splicing has been conducted in human 293 T cells [15] and, for a relatively small number of genes related predominantly to apoptosis and cancer, in cancer cells [16]. In this study, we sought to investigate the global impact of hnRNPH/F-mediated regulation of splicing events in oligodendrocytes and to determine whether genes involved in OL lineage progression are regulated by hnRNPH/F.

To this end, we have performed a genome-wide transcriptomic analysis at the gene and exon levels in Oli-neu cells treated with siRNA that target hnRNPH/F vs. untreated cells using Affymetrix exon array platforms. Gene expression levels and regulated exons were identified with the EASANA algorithm [17], [18]. Bioinformatics analyses were performed to determine the structural properties of G tracts, such as length, distance and position that correlate with the enhancing vs. silencing effect of hnRNPH/F. The expression of genes involved in signaling pathways was regulated by hnRNPH/F. Genes that regulate the transition of OPC to OL are differentially expressed in hnRNPH/F silenced Oli-neu cells. These changes were confirmed in developing OL *in vivo*.

This is the first genome wide analysis of splicing events and genes differentially regulated by hnRNPH/F in OL and the first report that hnRNPH/F regulate genes involved in the transition from OPC to OL.

## Materials and Methods

### Cell Cultures, Transfections and Primary Oligodendrocyte Cell Isolation

Oli-neu cells [19] were grown in SATO medium, as described [12], [19]. Oli-neu cells were transfected with 80 nM of siF/H, which targets both hnRNPH and F using the siPORT Amine reagents (Applied Biosystems) and cultured in growth medium for 72 hrs [12]. Total RNA was prepared and submitted for microarray analysis (Microarray Core Facility, University of Kentucky). Cell suspensions were prepared from the CNP-EGFP mouse brains (kind gift of Dr. V. Gallo) and EGFP^+^ OLs were isolated by Fluorescent Activated Cell Sorting (FACS) (FACS facility, University of Kentucky), as previously described [20], [21]. The animal work was approved by Institutional Animal Care and Use Committee at the University of Kentucky and was conducted in adherence with the University’s guidelines for animal husbandry.

### Affymetrix Exon Array Data Analysis

RNA was prepared using the RNeasy mini kit (Qiagen) from triplicate experimental and control mock siRNA treated Oli-neu cells. Five micrograms of total RNA were used to generate probes to hybridize with the Affymetrix Mouse Exon 1.0ST Array featuring ∼ 1 million exon clusters and 1.4 million probe sets (Microarray Core Facility, University of Kentucky). Since exon arrays contain multiple probes per exon, we were able to analyze both splicing and transcript levels.

Microarray dataset analysis and visualization were made using EASANA® (GenoSplice technology), which is based on the GenoSplice’s FAST DB® annotations [18]. Data were normalized using quantile normalization. Background corrections were made with antigenomic probes and probes were selected as described previously [22]. Only probes targeting exons annotated from FAST DB® transcripts were selected to focus on well-annotated genes whose mRNA sequences are in public databases [17]. Probes whose intensity signal was too low compared to antigenomic background probes with the same GC content were removed from the analysis. Only probes with a DABG *P* value ≤0.05 in at least half of the arrays were considered for statistical analysis [22]. Only genes expressed in at least one compared condition were analyzed. To be considered as being expressed, the DABG *P*-value had to be ≤0.05 for at least half of the gene probes.

We performed a paired Student’s t-test to compare gene intensities in the different biological replicates. Statistical analyses were also performed using the Student’s paired t-test on the splicing index to analyze the Exon Array data as described previously [22]. The splicing index corresponds to a comparison of gene-normalized exon intensity values between the two analyzed experimental conditions [22]. Exon and gene expression levels were classified in two groups indicated as high and low confidence. Bad-quality selected probes (*e.g.*, probes labeled by Affymetrix as ‘cross-hybridizing’) were removed from the analysis for the high confidence. For gene level analysis, genes were considered significantly regulated when fold-change was ≥1.5 and *P* value ≤0.05 for the high confidence and fold-change≥1.2, *P* value≤0.05 for the low confidence. Exons and part of exons were considered statistically significant for *P*-values ≤0.05 and fold-changes ≥1.2 for both high and low confidences.

### Pathway Analysis

Significant KEGG pathways [23] were retrieved using DAVID [24].

### RT-PCR and Real time qRT-PCR

Total RNA was extracted with the RNeasy mini kit according to the manufacturer’s instructions (Qiagen). The sequences of primers used for semiquantitative RT-PCR and for Real Time RT-PCR are shown in Table S1 and Table S2, respectively. qRT-PCR was performed using the StepOne™ real-time PCR system (Applied Biosystems) at the University of Kentucky Spinal Cord and Brain Injury Research Center core facility, as described [25], [26], and data was analyzed by the StepOne™ Software v2.0 (Applied Biosystems). Relative RNA levels were determined by comparing threshold cycles for individual RNA products normalized with GAPDH using the 2^−ΔΔCT^ method [27].

## Results

### hnRNPH and F Promote Both Exon Inclusion and Skipping

**Figure 1:**
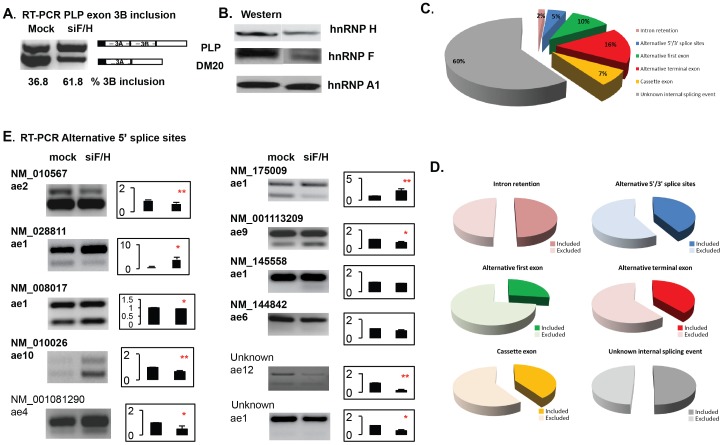
Genome wide analysis of alternative spliced events (ASEs) regulated by hnRNPH/F. Oli-neu cells were treated with an siRNA, siF/H that targets both hnRNPH and F [12]. RNA was used to generate probes to hybridize with the Affymetrix Mouse exon 1.0ST array featuring ∼ 1 million exon clusters and 1.4 million probe sets. We have analyzed splicing and transcript levels using the EASANA® from GenoSplice technology (www.genosplice.com). **A.** RT-PCR amplification of the endogenous PLP and DM20 transcripts. Schematic of the PCR products is shown. siF/H treatment induces a two-fold increase in the inclusion of exon 3B. Percent inclusion of exon 3B is shown. **B.** Western blot analysis of hnRNPH and F expression. More than 70% reduction of hnRNPH/F is induced by the siF/H treatment. hnRNPA1 is used as loading control. **C.** Pie chart showing the ASEs regulated by hnRNPH/F. Splicing of 252 exons was differentially regulated by knock down of hnRNPH/F. The types of spliced events are shown. **D.** Pie charts show the relative abundance of included (hnRNPH/F-repressed) and excluded (hnRNPH/F-activated) exons. For four of the six categories of alternative spliced events a greater number of exons are excluded (i.e. hnRNPH/F-activated) by depletion of hnRNPH/F. **E.** RT-PCR of alternative 5′ splice sites. Representative RT-PCR of 5′ASEs that were examined by semiquantitative RT-PCR analysis in siF/H treated vs. untreated Oli-neu cells (mock) (n = 3). Each ASE is labeled with the gene ID and the alternative spliced exon (ae) is shown. The ASEs that were validated by RT-PCR are shown in bold. The others, not bolded, demonstrated a change that was in the opposite direction of that detected in the arrays. Bar graphs represent the percent change of the exon inclusion ± SD in the siF/H treated cells vs. untreated cells set at 1 (n = 3). *≤0.05 and **≤0.01.

To investigate the global role of hnRNPH/F in the regulation of splicing events, we have performed a genome wide analysis of exon levels in Oli-neu cells that were treated with an siRNA, siF/H, which targets both hnRNPH and F, compared to mock siRNA treated cells. As previously published, treatment of Oli-neu cells with 80 nM siF/H reduces hnRNPH/F expression greater than 70% (Fig. 1B and [11]), which results in a two-fold increase in the PLP/DM20 ratio derived from the endogenous PLP transcript (Fig. 1A, [11]).

The exon levels were classified into high and low confidence groups (see Methods). Splicing of 252 exons was differentially regulated by knock down of hnRNPH/F in the high confidence group and 1,649 exons were differentially regulated by knock down of hnRNPH/F in the low confidence group. After manually inspecting the 252 exons from the high confidence group, individual types of alternatively spliced events were assessed. We found four intron retention (2%), twelve 5′/3′ ASEs (5%), 26 alternative first exon (10%), 41 alternative terminal exon (16%), 18 cassette exons (7%) and 151 unknown internal alternative splicing events (60%) (Fig. 1C, 1D).

The data show that hnRNPH/F regulate different types of alternative spliced events, indicating a broad role in the regulation of alternative splicing. In addition, in four of the six defined categories of ASEs, alternative first, alternative terminal,5′/3′ alternative splicing and cassette exons, there are more events that are excluded upon depletion (hnRNPH/F-activated) vs. those that are included (hnRNPH/F-repressed) upon depletion (Fig. 1D).

### Mechanisms of hnRNPH and F Mediated Regulation of ASE

In the case of 5′ alternatively spliced events, the proximal site is preferentially utilized [28], [29]. To gain insights into the mechanisms by which hnRNPH/F regulate selection of competing 5′ ss, we have conducted a thorough analysis of the 5′ ASEs that were identified in both the high and low confidence groups. We concentrated on the 5′ ASEs since the PLP alternative splicing is regulated by selection of competing 5′ ss through hnRNPH/F ([12] and Fig. 1A). We first validated fourteen (3 from the high confidence group and 11 from the low confidence group) 5′ ASEs by semiquantitative RT-PCR using primers that span the alternatively spliced exons (Table S1). For 3 hnRNPH/F-activated exons, we could not adequately amplify the alternative spliced products, hence we have further analyzed the remainder 11 5′ ASEs (data not shown). In three 5′ ASEs, the inclusion of the alternatively spliced exon is increased by knock down of hnRNPH/F, while in eight ASEs the inclusion is decreased (Fig. 1E). We confirmed the changes in exon inclusion/exclusion by RT-PCR in 73% (8 out of 11) of the ASEs (Fig. 1E, indicated in bold). These include one ASE in the hnRNPH/F-repressed exons and seven in the hnRNPH/F-activated exons (Fig. 1E). The changes in three ASEs (Fig. 1E, indicated in non bold characters) are in the opposite direction of that detected in the arrays. The data indicate that hnRNPH/F most often enhance the alternatively the spliced exon.

To determine whether the presence, position and length of the G tracts correlate with the outcome of splicing and allow a prediction of the effect mediated by hnRNPH/F, we have examined the G tracts in the alternatively spliced exon and in the downstream intron. Only three of the hnRNPH/F-activated ASEs did not have any G tracts in the downstream intron (Table S3). One (NM_183151, Mid1) could not be consistently amplified by RT-PCR, the other two were validated, however, for one (NM_144842, MYM type 5) the changes were not statistically significant while for the other (unknown gene, ae12) the changes were robust (see Figure 1E for RT-PCR results). The absence of G tracts suggests that these ASEs are not directly regulated by hnRNPH/F. In all the others, G tracts were present, suggesting a direct effect by hnRNPH and F.

The intron of the hnRNPH/F-activated exons contains on average five G tracts (1–11), of which 25% are quadruplets (11), 6% are quintuplets (3), 2% are sixtuplets (1) and the remainder is G triplets (Table S3). The intronic G tracts for the hnRNPH/F-repressed exons are on average 5 (3–6) of which 5% are quadruplets and quintuplets. Exonic G tracts are more represented in the hnRNPH/F-repressed exons (1–5), while fewer G tracts were present in the hnRNPH/F-activated exons (0–4) (Table S3). The differences in exonic and intronic G tracts between hnRNPH/F-activated and hnRNPH/F-repressed exons are statistically significant (p-value 1.29613E-19, Fisher’s exact test). Although the number of ASEs regulated by hnRNPH/F in the arrays is small, the data suggest that high density and length of the intronic G tracts and a relative paucity of exonic G tracts correlate with enhancement of the proximal 5′ ss by hnRNPH/F. In contrast, a balanced distribution of exonic and intronic G tracts is associated with inhibition of exon inclusion by hnRNPH/F. The latter may result from either silencing of the proximal 5′ ss or enhancement of the distal 5′ ss mediated by the exonic G tracts.

**Figure 2:**
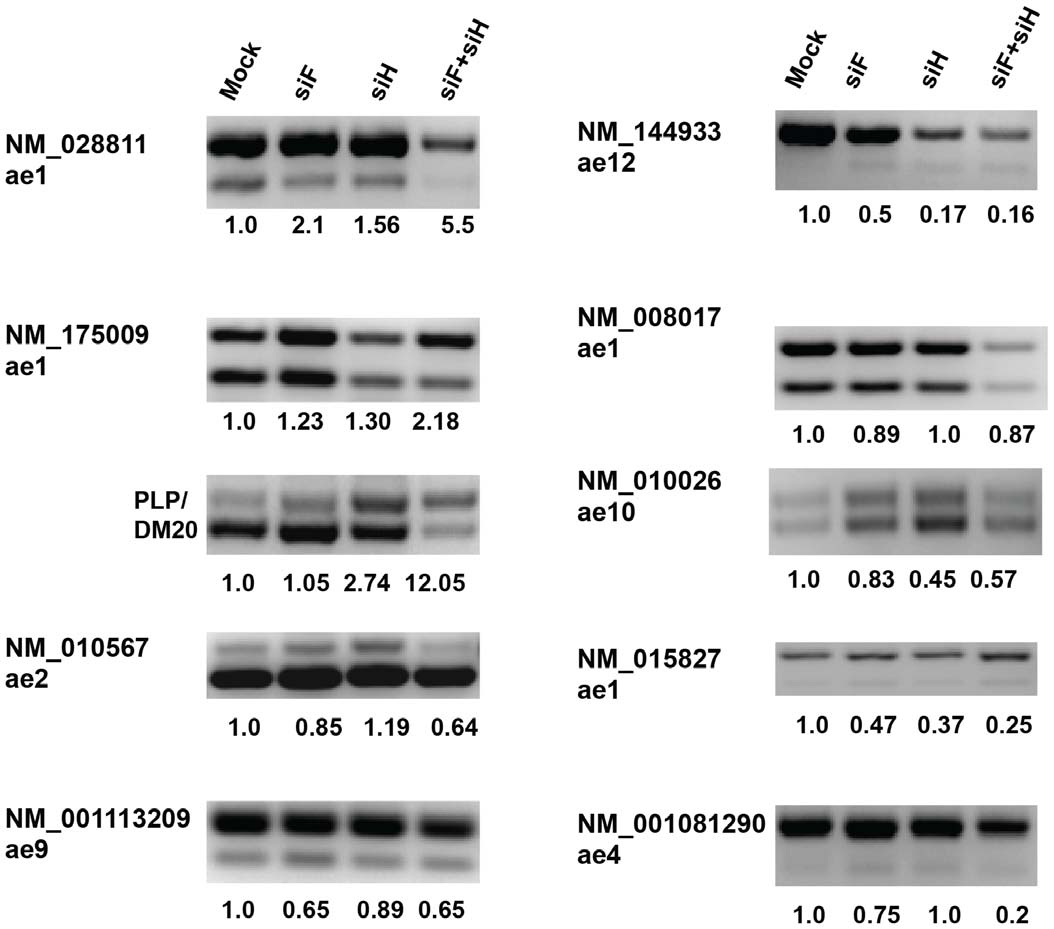
Changes in exon inclusion induced by silencing hnRNPH and F individually vs. both simultaneously. Representative RT-PCR (n = 2) of the products derived from 5′ASEs in Oli-neu cells treated with siRNAs that target hnRNPH (siH), hnRNPF (siF) or both (siF/H). We selected ASEs that were shown to have a statistically significant change in exon inclusion by RT-PCR. Mock are control untreated Oli-neu cells. Each ASE is labeled with the gene ID number and the ae is shown (also refer to Figure 1E). The number shown below each lane represents the fold change in exon inclusion compared to the mock treated cells set at the value of 1. The PLP/DM20 splicing event shows the synergistic effect of hnRNPH/F knock down.

We have shown that hnRNPH and F play distinct roles in the regulation of the PLP/DM20 ratio and cooperatively regulate the PLP exon 3B inclusion [11], [12]. Here, we sought to determine whether hnRNPH and F have a similar effect in the regulation of the 5′ ASEs identified in the arrays. For this analysis, we selected the ASEs whose changes were found to be statistically significant by RT-PCR analysis, as shown in Figure 1E. We have measured exon inclusion/exclusion for the 5′ ASEs by semiquantitative RT-PCR after silencing of hnRNPH and hnRNPF individually and compared it to the effect of silencing both. Importantly, knock down of either hnRNPH or F does not influence the abundance of the other as well as the expression of other hnRNPs [12], hence, the individual effect on the ASE can be measured separately in these reactions. The PLP/DM20 ASE was used as control (Fig. 2). The fold change in the inclusion of PLP exon 3B is 12 fold compared to 2 fold with siH and no change with siF, as previously shown [12]. For all ASEs, silencing hnRNPH and F individually had a modest effect and silencing both resulting in a greater change in the inclusion of the regulated exon, suggesting that in general they have a redundant function (Fig. 2).

In summary, hnRNPH and F most commonly enhance the inclusion of exons regulated by competing 5′ ss and this effect correlates with the density and length of intronic G tracts. Generally, hnRNPH and F are functionally redundant and their combined effect is additive.

### Gene Structure and G Tracts Determine the hnRNPH/F Regulation of ASEs

**Figure 3:**
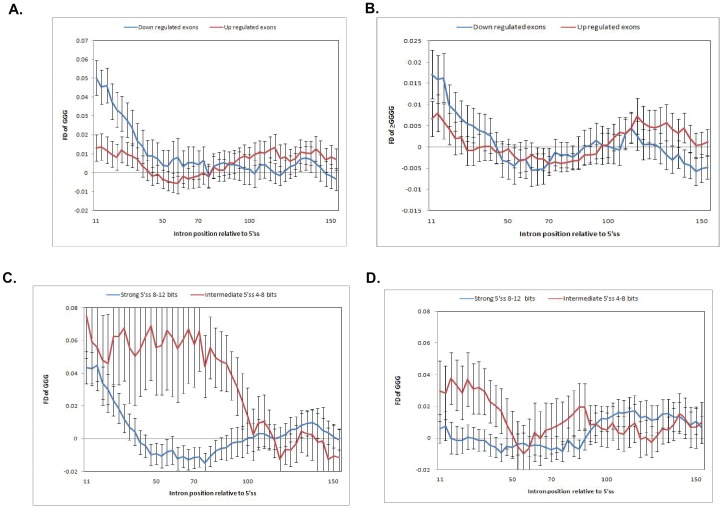
Frequency difference (FD) plot of G tracts in the intron downstream of internal cassette exons. A. FD plot of intronic G triplets for decreased inclusion (down regulated) and increased inclusion (up regulated) 5′ splice sites (ss). FD is defined as the difference between the observed frequency of GGG in introns, calculated in a 30-nt window, and the mean frequency of GGG in 10 random permutations of the sequence in the same window, with an offset of 3nt between successive windows, as described [10]. Black bars show the standard errors. **B.** FD plot of intronic G quadruplets and longer G tracts for down and up regulated 5′ ss. **C.** FD plot of G triplets in the intron downstream of intermediate and strong 5′ ss of exons with decreased inclusion (hnRNPH/F-activated). **D.** FD plot of G triplets in the intron downstream of intermediate and strong 5′ ss of exons with increased inclusion (hnRNPH/F-repressed).

Next, we sought to examine the G tract structural properties for internal exons, which account for 67% of the hnRNPH/F regulated ASEs in the arrays (in the high confidence group). We have performed a bioinformatics analysis of the distribution and length of intronic and exonic G tracts and correlated these features with the functional outcome. Of 169 internal exons (*i.e.*, cassette exon and/or unknown internal alternative events) inclusion of the regulated exon is reduced in 83 ASEs while it is increased in 86 ASEs. We have characterized G tracts in sequences extending from +11 to +150 of the intron downstream of the regulated 5′ ss and from −11 to −150 of the exon upstream of the regulated 5′ ss. We have excluded sequences between +1 and +10 since G tracts in this position overlap with the 5′ splice site and were shown to function as silencers [7] and enhancers [11]. The frequency difference (FD) plot of G triplets was calculated as previously described [10]. The highest FD is between +11 and +70 in the intron of both hnRNPH/F-repressed and hnRNPH/F-activated exons, however, the FD is 1–5 folds greater in hnRNPH/F-activated vs. -repressed exons (Fig. 3A). Furthermore, G quadruplets or longer G tracts are more commonly clustered between +11 and +40 and are more abundant in hnRNPH/F-activated exons (Fig. 3B). Additionally, G runs are within 30 nucleotides from the 5′ ss in 44 of the hnRNPH/F-activated exons compared to 20 of the hnRNPH/F-repressed exons (Table S4). G runs are positioned closer to each other, especially the first and second G run, are separated by < = 20 nucleotides and contain longer runs of Gs in the hnRNPH/F-activated vs. –repressed exons (Table S4). The FD plot of G triplets in the upstream exon sequences shows an overall lower G triplet representation in both hnRNPH/F-activated and -repressed 5′ ss without significant differences between the activated vs. repressed exons (data not shown). Together, the data show higher density and length of G runs in the intron downstream of the hnRNPH/F-activated exons, consistent with their role as ISE.

Next, we have examined the G tract dependence on the 5′ ss strength and the functional outcome on the regulated exon. Greater clustering of intronic G triplets occurs at intermediate 5′ ss (4–8 bits) vs. strong 5′ ss (8–12 bits) for hnRNPH/F-activated and -repressed exons considered as a group (Fig. S1). The FD of G triplets was higher for hnRNPH/F-activated vs. hnRNPH/F-repressed exons for both intermediate and strong 5′ss (Fig. 3C and 3D). However, the FD of intronic G triplets was 2–8 fold greater for intermediate 5′ ss compared to strong 5′ ss (Fig. 3C and 3D), in keeping with the dependence of ISE activity on the strength of the 5′ ss [7].

The data indicate that the density and length of the G tracts within the first 70 nucleotides of the intron is associated with hnRNPH/F-dependent enhancement of the upstream 5′ ss. This effect is independent from the strength of the 5′ ss, although the clustering of intronic G tracts is higher for intermediate 5′ ss, in keeping with an evolutionarily conserved role of G tracts as enhancers of weak 5′ splice sites [7]. The presence of G tracts in the exonic sequences does not show a clear association with either an enhancer or silencer effect by hnRNPH/F.

### hnRNPH and F Regulate Expression of Genes Involved in OL Differentiation

Knock down of hnRNPH/F affected 6% of the expressed genes in the low confidence group (832 transcripts out of 12,948 expressed genes). Of the regulated genes, 23% (188) were more expressed and 77% (644) were less expressed in the low confidence group. Among the 832 regulated genes, 131 (“low confidence group”) (16%) also gather at least one differentially regulated exon, while of the 12,948 expressed genes, 1,204 (9%) gather at least one differentially regulated exon (“low confidence group”). The data show an enrichment of differentially regulated exons in the genes whose expression is affected by silencing hnRNPH/F.

**Figure 4:**
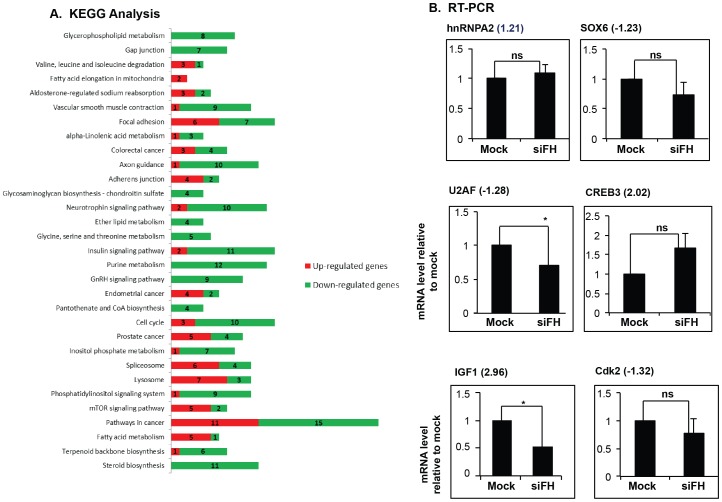
Genes are differentially regulated at the transcriptional levels. **A.** Representation of significant pathways whose genes are affected by knock down of hnRNPH/F. Thirty one Kegg pathways were significantly affected. Genes are either up- or down-regulated. **B.** Changes in gene expression were verified by Real Time RT-PCR in Oli-neu cells depleted of hnRNPH and F. Bar graphs represent the mean±SD of transcript levels of the indicated genes quantitated by Real Time RT-PCR in mock siRNA treated (Mock) and siF/H treated Oli-neu cells (n = 3). Oli-neu cells were treated with siF/H and harvested after 72 hrs in culture for Real Time RT-PCR analysis. The data are expressed as percent change of the treated vs. mock cells, the latter is set at the value of 1. ns = non significant, *p = 0.05. In parenthesis next to the gene name is shown the fold change in the microarrays.

A goal of this study was to determine whether hnRNPH and F control the expression of genes that are important for OL cell biology. By KEGG pathway analysis [23], we found that there was an enrichment of genes that are involved in the insulin-IGF signaling pathway, mTOR pathway, RNA binding proteins and cell cycle (Fig. 4A). Because of the relevance of these pathways in OL lineage progression, we have selected a number of genes and validated the expression changes by Real Time qRT-PCR in Oli-neu cells after knock down of hnRNPH/F. We have selected 15 genes involved in OL lineage progression (Table S5). We have validated approximately 60% of the differentially expressed genes (Fig. 4B and Table S5). IGF1 level was found to be significantly increased in the arrays, but was found to be decreased by Real Time qRT-PCR analysis (Fig. 4B). Other genes that are involved in cell cycle progression (cdk2) and negative regulator of OPC differentiation (SOX6) were reduced both in arrays and by Real Time qRT-PCR analysis (Fig. 4B). Splicing of a constitutive exon is differentially regulated by depletion of hnRNPH/F along with changes in expression of cdk2, SOX6 and hnRNPA2/B1, suggesting that an effect in splicing may be coupled with the change in expression (Table S5 and Discussion). In support of this possibility, CLIP-Seq tags were identified in cdk2 [7], while SOX6 was shown to be regulated by hnRNPF [15].

**Figure 5:**
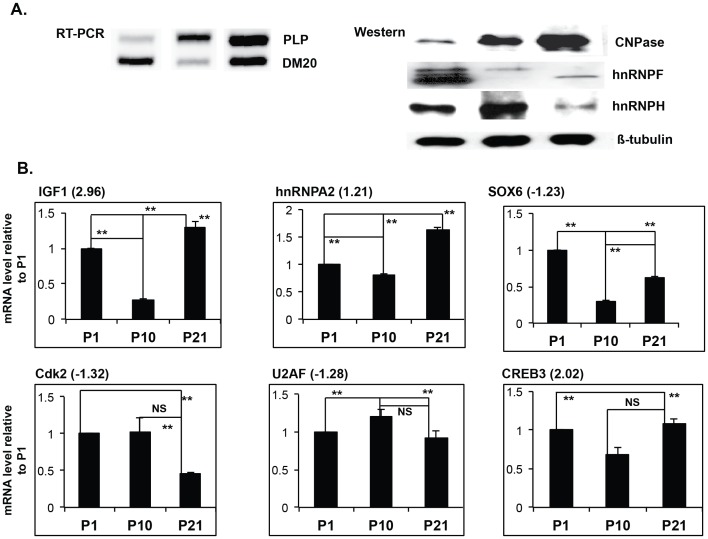
Expression profile of the validated genes in developing oligodendrocytes *in vivo*. CNPase-EGFP+ oligodendrocytes were isolated at post-natal day (P) 1, 10 and 21 [43] and subjected to RT-PCR, Western blot analysis and Real Time RT-PCR. **A.** Representative RT-PCR of PLP/DM20 ratio and Western blot analysis of hnRNPH and F in developing oligodendrocytes. CNPase is a marker of differentiation and increases in P10 and P21 OL vs. P1 oligodendrocyte progenitor cells. β-tubulin is the loading control. **B.** Bar graphs represent the mean ± SD of transcript levels of the indicated genes quantitated by Real Time RT-PCR in EGFP+ oligodendrocytes *in vivo* (n = 3). The fold change detected in the microarray is shown in parenthesis next to the gene name. ns = non statistically significant. **≤0.01.

To demonstrate the biological relevance of the gene expression changes induced by hnRNPH/F knock down, we have quantified their expression in developing OL *in vivo*. We have isolated OPC, pre-OL and differentiated OL by sorting EGFP+ oligodendrocyte lineage cells at post-natal day 1, 10 and 21 respectively, as described [21]. We have quantitated the expression levels of IGF1, which plays a pivotal role in OL survival and differentiation [30], SOX6, which is a negative regulator of OL differentiation [31], CREB3 which is a critical transcription factor and a target of the differentiation inducing cAMP pathway [32], cdk2, which regulates cell cycle progression in OL terminal differentiation [33], [34], hnRNPA2/B1, which regulates transport and translation of the myelin basic protein in differentiated OL [35], [36] and U2AF which is an essential spliceosomal factor [37]. The expression of IGF1, CREB3 and hnRNPA2/B1 increases in p21 day OL, while levels of SOX6 and cdk2 decrease at p21 vs. p1 OPC (Fig. 5B). U2AF, a spliceosomal factor is lower in p21 OL. The changes in the expression of these genes in p21 OL vs. P1 OL were similar to those detected by arrays, the fold change in the arrays is indicated in parenthesis in Figure 5. The expression levels in p10 OL showed different patterns for each gene examined. In the case of IGF1 and hnRNPA2/B1, there was a decrease at p10 compared to an increase at p21, cdk2 expression did not change at p10, but was significantly decreased at p21, while SOX6 was drastically decreased at p10. The data suggest that these genes have distinct temporal regulation reflecting different roles in development.

Differential expression of these genes in p21 OL is associated with a dramatic reduction in the abundance of hnRNPH and F in p21 OL vs. p1 OPC (Fig. 5A). Furthermore, the fold change of the selected genes measured in the arrays is similar to those that occur *in vivo*. Notably, IGF1 is up-regulated in the arrays and in differentiated p21 OL, however, it was reduced at p10, similarly to the results in Oli-neu cells treated by siF/H (compare Fig. 4B with Fig. 5B).

In summary, knock down of hnRNPH/F reduces the expression of negative regulators of OL differentiation while it increases the expression of differentiation inducing genes, replicating changes that occur in developing OL *in vivo*. The data suggest that hnRNPH/F may participate in the regulation of OL lineage progression.

## Discussion

Our study is the first to examine the global impact that knock down of hnRNPH and F has on alternative splicing and gene expression in a brain derived oligodendrocytic cell line. We show that hnRNPH/F regulate different types of alternative splicing and the most common outcome caused by depletion of hnRNPH/F is reduced inclusion of the alternatively spliced exon suggesting a role as enhancers except for internal exons. In addition, our studies have uncovered a previously unrecognized function of hnRNPH/F in the regulation of gene expression. Importantly, a significant number of differentially expressed genes are components of signaling pathways that regulate differentiation of oligodendrocytes, hence, positioning hnRNPH/F in the regulatory network that control oligodendrocyte lineage progression.

Although hnRNPH/F are typically considered repressors of alternative splicing, we show that knock down of both results in decrease of the exon inclusion for four of the six categories of spliced events examined consistent with enhancer function. We conducted a careful analysis of the distribution, density and length of the G runs in the intron downstream and the exon upstream of the regulated 5′ ss. hnRNPH/F regulated genes have a higher frequency distribution of intronic G triplets in keeping with the known function of G triplets as the basic binding motif for hnRNPH/F and the G run ISE activity [7]. In both internal exons and 5′ alternative spliced events, there is a greater clustering of G triplets and a higher representation of longer G tracts in the intron downstream of the regulated 5′ ss in genes that are enhanced vs. those that are repressed by hnRNPH/F. The relative higher density of intronic G triplets, especially between +11 and +30 nucleotides, in genes enhanced vs. repressed by hnRNPH/F may reflect differences in the mechanism by which hnRNPH/F activate vs. repress splicing.

We have concentrated on the 5′ alternative spliced events as this is the mechanism that regulates alternative splicing of the PLP gene by hnRNPH/F in oligodendrocytes. High density and length of the intronic G tracts and a relative paucity of exonic G tracts correlate with enhancement of the proximal 5′ ss by hnRNPH/F. In contrast, a balanced distribution of exonic and intronic G tracts is associated with inhibition of exon inclusion. These results are consistent with the knowledge that intronic G runs function as ISE, while exonic G runs function as silencers [38].

The higher G peaks close to the 5′ ss and the greater density close to weak 5′ ss was previously reported in genome wide studies [7], [9], [10]. Our study shows that there is a selective clustering of G runs associated with the role of hnRNPH/F as enhancers vs. a function as repressors. How might the higher density of G runs and the greater length favor the role of hnRNPH/F as enhancers? Clustering close to the 5′ ss is thought to reflect an optimal distance required for the splicing factors to interact with the spliceosome [10]. Interestingly, binding of hnRNPH/F to G runs through the qRRMs prevents formation of G mediated RNA secondary structure [39]. Upon binding of hnRNPH/F, the higher number of G runs may generate a more “open” RNA structure which enhances recognition of the 5′ ss by the spliceosome. Alternatively, the G runs may favor the interaction of hnRNPH/F with the spliceosome and enhance either the ATP-independent [13] or the ATP-dependent steps of spliceosomal assembly [40].

The greater representation of genes that are enhanced by hnRNPH/F in some categories of alternatively spliced events led us to conclude that hnRNPH/F more frequently activate exon inclusion in these events. It is possible that this result represents a skewed detection in our arrays on the basis of a stronger effect on genes that are enhanced vs. those that are inhibited by hnRNPH/F. However, we think that this is unlikely. Our data are in keeping with those reported by a genome wide analysis of human cells showing that hnRNPH1, F, M and U activate exon inclusion for a majority of types of ASEs [15]. Furthermore, knock down of hnRNPH/F also reduced inclusion of the alternatively spliced exon of most apoptotic genes examined by RT-PCR in cancer cells [39]. Interestingly, none of the alternatively spliced events examined in the latter study were significantly changed in our microarrays. The absence of overlaps with splicing events identified in that study may reflect the nature of the cells and the methodologies used in each study. We have performed the analysis in non cancer cells and we have used microarrays that have a different dynamic range than the RT-PCR detection method used in the other study.

We have also examined the effect of depletion of both hnRNPH/F vs. depletion of each individually. We were interested in determining whether a cooperative effect similar to that we described for PLP is utilized for other 5′ ASE. We show that for the 5′ ASEs that displayed a significant change upon knock down of both hnRNPH/F, the effect of hnRNPH and F is additive and their function is redundant as the individual knock down has a smaller effect or no effect. It remains to be determined what elements in the gene structure would determine this outcome vs. the cooperative effect active in the PLP gene. An important difference is that PLP is a cell-specific ASE, while the 5′ASEs identified in the arrays are not, suggesting that cell specific factors may contribute to the synergism.

A novel finding of our study is that 6% of genes are regulated at the expression level with two thirds being down regulated by knock down of hnRNPH/F. Interestingly, 16% were also regulated at the exon level compared to 9% of the total expressed genes, suggesting a splicing dependent regulation of gene expression. Additionally, hnRNPH/F may regulate gene expression by an RNA mediated mechanism by affecting message stability through binding to G runs in the 3′ UTR especially close to polyA sites [41], [42]. Interestingly, the 3′ UTRs of the genes examined contain multiple G triplets and long 4–6 G tracts, suggesting that a 3′ UTR-mediated regulation may play a role (data not shown). Another possibility is that depletion of hnRNPH/F causes a change in the expression of other splicing factors. Cross-regulation of hnRNPs was recently demonstrated in human cells after depletion of individual hnRNPs [15], pointing to a complex regulatory network. However, we did not detect significant changes of other hnRNPs in our arrays, with the exception of hnRNPA2/B1. Changes in the expression of SR proteins, Sfrs7, Sfrs11 and Tra2α, were detected by arrays, but could not be confirmed by subsequent Real Time qRT-PCR in Oli-neu cells depleted of hnRNPH/F (data not shown).

Of the genes examined and involved in OL differentiation, regulation at the exon level was detected for cdk2, SOX6 and hnRNPA2/B1 after depletion of hnRNPH/F, suggesting that changes in splicing may result in changes in transcript levels/stability. For the other genes, hnRNPH and F may regulate transcription, possibly indirectly by affecting genes involved in transcriptional regulation. An important conclusion of the results is that expression of genes that inhibit OL differentiation, cdk2 and SOX6, is reduced by knock down of hnRNPH/F while genes that promote OL differentiation, CREB and IGF1 are increased. In addition, the increased expression of IGF1 suggests a possible autocrine mechanism by which OL may regulate lineage progression.

These data suggest that hnRNPH/F might regulate OL proliferation and differentiation. In support of this statement, we show that the expression of hnRNPH/F decreases in developing OL *in vivo* at the time of the other gene expression changes. Interestingly, hnRNPH and F decrease according to a distinct temporal pattern suggesting that each factor may serve independent functions. Importantly, IGF1 and hnRNPA2/B1 demonstrated a biphasic pattern of expression, i.e. high at p21, when both hnRNPH and F are decreased and low at p10, when hnRNPH is still expressed, compared to p1, suggesting a differential role of each hnRNPH and F on these genes. The expression of other genes changed in the same direction at p21 and p10. These patterns are likely to reflect their individual roles in OL lineage progression, the mixed nature of OL development at p10 and/or be influenced by the different temporal course of hnRNPH/F decreased expression. Future studies will investigate the role of hnRNPH and F in gene regulation in developing OL.

In summary, our studies show that hnRNPH/F exert a broad effect on regulation of splicing and gene expression in OL.

## Supporting Information

Figure S1 **Clustering of intronic G triplets downstream of intermediate and strong 5′ ss.** Frequency difference (FD) plot of G triplets in the intron downstream of intermediate and strong 5′ ss in both hnRNPH/F-activated and -repressed exons.
(TIF)

Table S1 **Sequences of the RT-PCR primers.** The sequences of the forward and reverse primers used for RT-PCR are shown and labeled by the gene number and in parenthesis by the gene name, when available, for the 14 ASEs that were analyzed by RT-PCR. For the unknown genes we indicate in parenthesis the alternatively spliced exon (ae).
(DOC)

Table S2 **Sequences of Real Time qRT-PCR primers.** The sequences of the forward and reverse primers used for Real Time qRT-PCR are shown and labeled by the gene ID number. In parenthesis is shown the gene name.
(DOC)

Table S3 **Analysis of G tracts in the exon upstream and the intron downstream of the regulated 5′ ASEs.** We show the sequence, position and length of the G tracts in the exon upstream and intron downstream of the regulated 5′ splice site for the fourteen ASEs analyzed by RT-PCR. The G tracts are color labeled depending on the length of the G run. For each ASE, we show the gene ID number, gene symbol and whether hnRNPH and F activate or repress.
(XLS)

Table S4 **G tract analysis in the exon upstream and intron downstream of the regulated 5′ splice site for internal exons.** The Table shows the position, sequence and length of exonic and intronic G tracts for 190 exons whose splicing is affected by depletion of hnRNPH/F. Twenty one are alternative first exons and one hundred and sixty nine are internal exons (cassette and unknown). We show the gene ID number, gene name, the regulated exon and whether the exon is down- or up-regulated. The G tracts are color labeled depending on the length of the G run.
(XLSX)

Table S5 **List of genes with biological relevance for oligodendrocytes and regulated by hnRNPH and F.** We show the ID number and name of genes that are relevant to oligodendrocyte cell biology and whose transcript levels were verified by Real Time qRT-PCR in siF/H treated compared to control treated Oli-neu cells (n = ≥2). Approximately sixty percent of the expression changes was confirmed by Real Time RT-PCR (shown in bold). We indicate the genes for which a change in exon splicing was also detected by array upon depletion of hnRNPH/F.
(DOC)
