# Supplementary figures and images for: Global Profiling of Alternative Splicing Events and Gene Expression Regulated by hnRNPH/F
Source: PLoS One. 2012 Dec 17;7(12):e51266. doi: 10.1371/journal.pone.0051266 (PMC3524136; doi:10.1371/journal.pone.0051266)

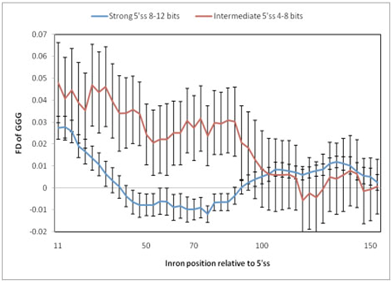

Supplement: Figure S1 — Clustering of intronic G triplets downstream of intermediate and strong 5′ ss. Frequency difference (FD) plot of G triplets in the intron downstream of intermediate and strong 5′ ss in both hnRNPH/F-activated and -repressed exons. (TIF) [file pone.0051266.s001.tif]
