# Supplementary material for: Global Profiling of Alternative Splicing Events and Gene Expression Regulated by hnRNPH/F
Source: PLoS One. 2012 Dec 17;7(12):e51266. doi: 10.1371/journal.pone.0051266 (PMC3524136; doi:10.1371/journal.pone.0051266)
Supplement: Table S1 — Sequences of the RT-PCR primers. The sequences of the forward and reverse primers used for RT-PCR are shown and labeled by the gene number and in parenthesis by the gene name, when available, for the 14 ASEs that were analyzed by RT-PCR. For the unknown genes we indicate in parenthesis the alternatively spliced exon (ae). (DOC) [file pone.0051266.s002.doc]

**Table S1**

**NM_008017 (SMC2)**

Forward GGTTGTTGGCTGCAGCGGAT

Reverse ACTGTCTTGGGTTAGTAAGCCTGT

**NM_028811 (Elp3)**

Forward GCTAAGTTTCTCCGCCTTCTAAAC

Reverse CAACCAGCTGCTTGATAACGTCTC

**NM_010567 (Inppl1)**

Forward GCTAGGAGTCAACCCGAGTCGC

Reverse CGGGGAAGCTTGAAGTCGAGGT

**NM_001081290 (BAT2 domain)**

Forward CAGAAGTGTCACCAGCACAGCCCA

Reverse AGCTGCCTGCAGACTGGGAAATTC

**NM_010026 (Ddef1)**

Forward ATAAAGCAAACCCAAGATGAAGA

Reverse CATCACTCTTCTTCAGCAGGA

**NM_144842 (MYM type 5)**

Forward CTATGAAAACTGCCAGAGTCTTAG

Reverse GCTACACTGTTCACAGCAGTTCAT

**NM_145558 (Hadhb)**

Forward GTCAGTCTGGACTTGACCTTGTTG

Reverse AAGTGGAAGTCAAGATGGTAGTCAT

**NM_001113209 (Nf1b)**

Forward TGCAGTGCGACCTGTGACCCTGAC

Reverse CGATTGGCTTGAGATGTGCCTGAG

**NM_175009 (Eny2)**

Forward GGTGCCCGAGCTACTGAGGGTCTA

Reverse GCTGCTCTCATCTGCGCATCT

**Unknown (ae1)**

Forward GGTCTGAGCTACTGGAACTC

Reverse CATCCGTGGTGAGAAAAGGCAGGA

**Unknown (ae12)**

Forward ATTGTTGTGCTGCTCATGTCCATG

Reverse CAGTTAGAGAGAGAGTCTGTAGA

**Unknown (ae1)**

Forward AGACCATGGGGAGGTGCTAACTTC

Reverse TTCAGAGAGGCATTGAAGATGATG

**NM_183151 (Mid1)**

Forward GGCTTCCAGAGCGTCGTGACATCT

Reverse TGTGAGTGATTTTCTGAG

**Unknown (ae3)**

Forward GAGTACTTTACTAAGCGAAGGAGAT

Reverse CCTCAGATGCTGAACATATGCTGA
