# Supplementary material for: Global Profiling of Alternative Splicing Events and Gene Expression Regulated by hnRNPH/F
Source: PLoS One. 2012 Dec 17;7(12):e51266. doi: 10.1371/journal.pone.0051266 (PMC3524136; doi:10.1371/journal.pone.0051266)
Supplement: Table S2 — Sequences of Real Time qRT-PCR primers. The sequences of the forward and reverse primers used for Real Time qRT-PCR are shown and labeled by the gene ID number. In parenthesis is shown the gene name. (DOC) [file pone.0051266.s003.doc]

**Table S2**

**NM_010207 (fgfr2)**

Forward AGGCATGGAGTACTTGGCTTCCCA

Reverse TAGTAGTCTATGTTGTTGATATCCCT

**NM_019963 (stat2)**

Forward TTAATTTTGAAGAACAGAGGAAATATTTGA

Reverse CAAGAGCTCTGCATTCACTTCTAAG

**NM_015733 (casp9)**

Forward CCTGCTCGGCTCAAGCCAGAGGTT

Reverse GCATGTCCCCTGATCTTCCCTGGA

**NM_133671 (u2af)**

Forward CTACTCTTCCCAAGTATAAGGTCT

Reverse TTATTGGGCAAGGAATAAGGGATC

**NM_013822 (Jag1)**

Forward TGGGATTCCAGTAATGACACTATTC

Reverse TCTGCCATTGCCGGTAGGGTTTA

**NM_013497 (creb3)**

Forward TTCCTCTCACTAAGGTGGAGGAACAAG

Reverse TCCAGCCCCACGACATACACCTTCT

**NM_009716 (atf4)**

Forward TAAGCCATGGCGCTCTTCACGAAATCCA

Reverse TCGGTCATGTTGTGGGGCTTTGCT

**NM_011445 (Sox6)**

Forward GGTCACATGCCTCCGCTCATGATC

Reverse TTGTGGGTGGGAGACATGACCCTT

**NM_010512 (igf1)**

Forward CTTGCTCACCTTCACCAGCTCCAC

Reverse TGGAGCCATAGCCTGTGGGCTTGT

**NM_016806 (hnRNPA2/B1)**

Forward GCTGTAGCAAGAGAGGAGTCTGGA

Reverse TCCATACTCTTCAAAGTAATCTCTAAG

**NM_183417 (cdk2)**

Forward CTCTTCCCCTCATCAAGAGCTATC

Reverse CAAGATGGACCCCTCTGCATTGATA

**NM_145990 (cdk5rap2)**

Forward ACAAGTTCCAAGACCTCTCGCCAT

Reverse CTCATGTGTTGACGGTAAATGACACA

**NM_010411 (hdac3)**

Forward CTGAAGAGAGAGGTCCCGAGGAGA

Reverse GCTGCTCtAAATCTCCACATCACTTTC

**NM_010127 (Pou6f1)**

Forward CGACCATTCTGCAGAGGCAACTG

Reverse GGGGCTTGGTTGAAGGTGGCAATC

**NM_001093753 (Sfrs11)**

Forward CAACCCACTTACCCAGATTGGCGCT

Reverse TTCCTGGAAGCCCAAGAGCAGCAAG
