# Supplementary material for: Global Profiling of Alternative Splicing Events and Gene Expression Regulated by hnRNPH/F
Source: PLoS One. 2012 Dec 17;7(12):e51266. doi: 10.1371/journal.pone.0051266 (PMC3524136; doi:10.1371/journal.pone.0051266)
Supplement: Table S5 — List of genes with biological relevance for oligodendrocytes and regulated by hnRNPH and F. We show the ID number and name of genes that are relevant to oligodendrocyte cell biology and whose transcript levels were verified by Real Time qRT-PCR in siF/H treated compared to control treated Oli-neu cells (n = ≥2). Approximately sixty percent of the expression changes was confirmed by Real Time RT-PCR (shown in bold). We indicate the genes for which a change in exon splicing was also detected by array upon depletion of hnRNPH/F. (DOC) [file pone.0051266.s006.doc]

**Table S5**

| **Gene ID** | **Gene name** | **Microarray**  **Expression /exon splicing** | **Real Time RT-PCR**  **Expression** |
| --- | --- | --- | --- |
| NM_010207 | (FGFR2) | Up | down |
| NM_019963 | (Stat2) | down | up |
| NM_015733 | (Casp9) | down | Up |
| **NM_133671** | **(U2AF)** | **down** | **down** |
| **NM_013822** | **(Jag1)** | **Down/exon7** | **down** |
| **NM_013497** | **(CREB3)** | **Up** | **up** |
| **NM_009716** | **(Atf4)** | **Down** | **down** |
| **NM_011445** | **Sox6** | **Down/exon8** | **down** |
| NM_010512 | (Igf1) | Up | Down |
| **NM_016806** | **(hnRNPA2/B1)** | **Up/exon1** | **Up** |
| **NM_183417** | **Cdk2** | **Down/ exon6** | **Down** |
| NM_145990 | (cdk5rap2) | Up | down |
| **NM_010411** | **(hdac3)** | **Down** | **Down** |
| NM_010127 | (Pou6f1) | Down | Up |
| NM_001093753 | (Sfrs11) | Up/exon4 | Down |
